# *Moraxella nonliquefaciens* bloodstream infection and sepsis in a pediatric cancer patient: case report and literature review

**DOI:** 10.1186/s12879-019-4489-y

**Published:** 2019-10-11

**Authors:** Carlos L. Correa-Martínez, Kerstin K. Rauwolf, Franziska Schuler, Miriam Füller, Stefanie Kampmeier, Andreas H. Groll

**Affiliations:** 1 Institute of Hygiene, University Hospital Münster, Robert-Koch-Strasse 41, 48149 Münster, Germany; 2 Department of Pediatric Hematology and Oncology and Center for Bone Marrow Transplantation, University Children’s Hospital Münster, Münster, Federal Republic of Germany; 3 Institute of Medical Microbiology, University Hospital Münster, Münster, Germany

**Keywords:** Sepsis, *Moraxella nonliquefaciens*, Children, Infection, Cancer

## Abstract

**Background:**

*Moraxella nonliquefaciens* is a usually non-pathogenic biofilm-producing Gram*-*negative coccobacillus which may colonize the upper respiratory tract, rarely causing invasive disease. Although very rare, bloodstream infections caused by this organism have been described, showing often a fatal outcome. Here, we report the case of a pediatric cancer patient with bloodstream infection and sepsis due to *M. nonliquefaciens* showing full recovery after appropriate antibiotic treatment.

**Case presentation:**

A three-year-old boy with stage IV neuroblastoma was admitted for high-dose chemotherapy with autologous stem cell rescue after standard neuroblastoma treatment. Despite receiving antimicrobial prophylaxis with trimethoprim/sulfamethoxazole, acyclovir and amphothericin B, the patient presented with fever of up to 39.5 °C and neutropenia. Besides a chemotherapy-related mucositis and an indwelling Broviac catheter (removed), no infection focus was identified on physical examination. *Moraxella nonliquafaciens* was identified in blood cultures. After antibiotic treatment and neutrophil recovery, the patient was fit for discharge.

**Conclusions:**

The case described highlights the importance of an otherwise non-pathogenic microorganism, especially in immunosupressed cancer patients. It should be kept in mind that, although very infrequently, *Moraxella nonliquefaciens* may cause bloodstream infections that can be successfully treated with prompt focus identification and antibiotic therapy.

## Background

*Moraxella nonliquefaciens* is a Gram-negative coccobacillus that may be found in the upper respiratory tract as part of the local flora [1], rarely causing disease. Cases of localized infection have been described, including meningitis [2], endophthalmitis [3, 4], endocarditis [5–7] and pneumonitis [8]. To the best of our knowledge, only four cases of bloodstream infection with this pathogen have been reported to date (Table 1) [9–12], only two of them in hemato-oncological patients [9, 10] who subsequently died. Here, we present the first case of bloodstream infection and sepsis by *M. nonliquefaciens* in a neutropenic pediatric cancer patient showing full recovery following central venous catheter removal and appropriate antibiotic treatment.

**Table 1:** Summary of cases of *Moraxella nonliquefaciens* bloodstream infections reported in the literature

Case	Reference, year	Age (years), gender	Underlying condition	Involved organs/sites	Antibiotic treatment, duration (days)	Catheter handling	Outcome
*Hemato-oncological patients*							
1	Brorson, 1983 [9]	75, f	Multiple myeloma	Blood	Gentamicin, Penicillin, 6	Not specified	Recovery
2	Mongkolrattanothai, 2018 [10]	1, f	Multisystem Langerhans cell histiocytosis (LCH), neutropenia	Blood	Not specified	Not specified	Short recovery, readmission after 4 days with subsequent death due to disseminated cryptococcosis
3	Correa-Martínez, 2019 (present case)	3, m	Stage IV neuroblastoma, neutropenia	Blood	Piperacillin/ tazobactam plus gentamicin followed by meropenem plus teicoplanin, 11	Removal of the indwelling Broviac catheter	Recovery
*Non-hemato-oncological patients*							
4	Sharma, 1974 [11]	0.5, m	Recurrent respiratory tract infections.	Blood, urinary tract	Penicillin, < 1	Not specified	Death
5	Kavkalo, 1985 [12]	44, f	Cicatricial esophageal stenosis, subsequent gastrostomy.	Blood, abdominal cavity	Not specified	Not specified	Death

## Case presentation

We present the case of a three-year-old boy with stage IV neuroblastoma admitted for high-dose chemotherapy with treosulfan (12 g/m^2^ × 3 days) and melphalan (140 mg/m^2^ × 1 day) with autologous stem cell rescue for consolidation after multimodal treatment consisting of intensive chemotherapy, surgery, and I^131^-metaiodobenzylguanidine therapy according the recommendations of the German Neuroblastoma Registry 2016 [13]. Antimicrobial prophylaxis included trimethoprim/sulfamethoxazole (4 mg/m^2^ BID on 2 days per week until day − 1), acyclovir (5 mg/kg TID) from day − 1 onward and amphotericin B (100 mg TID, oral suspension).

**Fig. 1:**
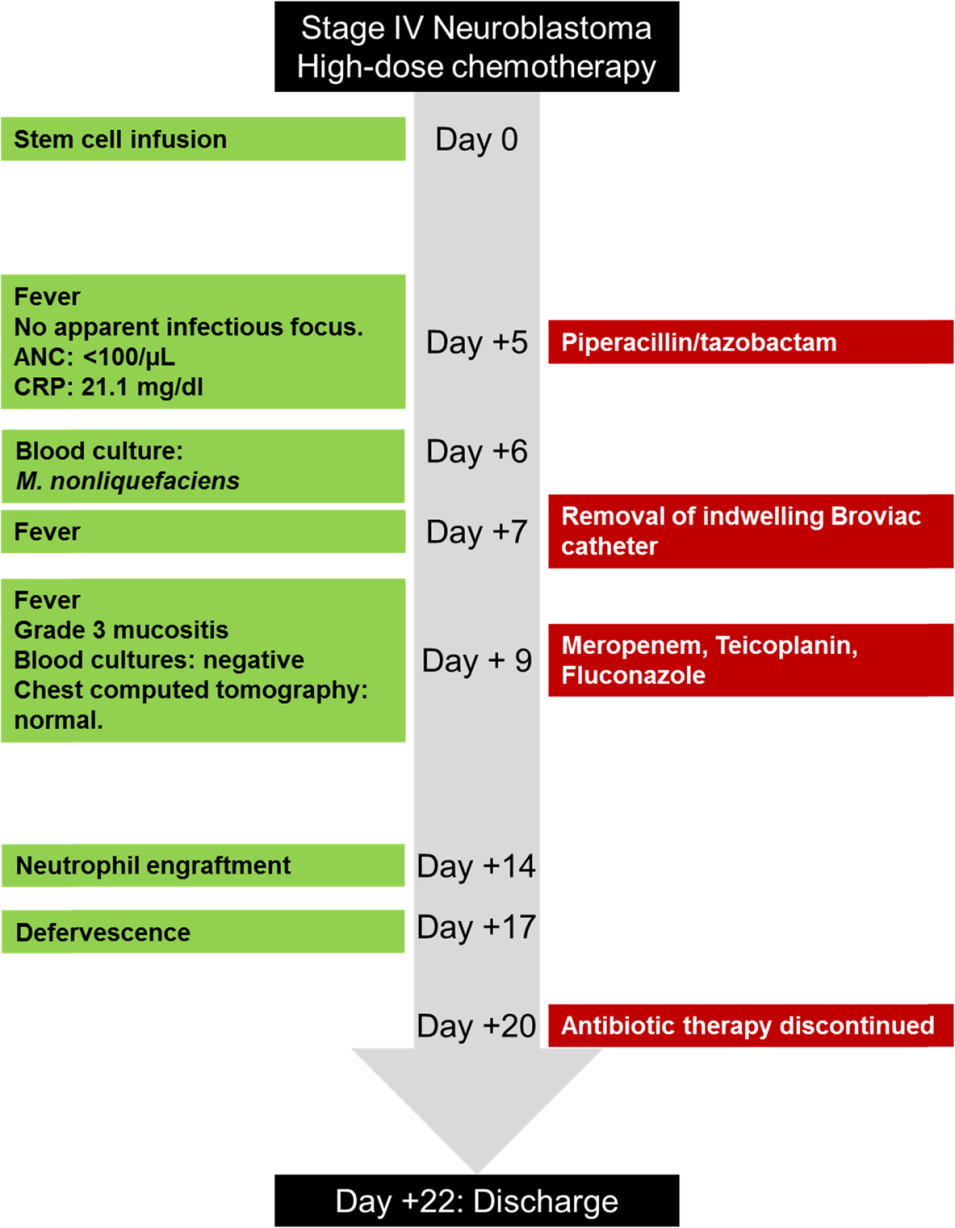
Timeline of the patient’s clinical course

Five days following stem cell infusion (day + 5), the patient presented fever of up to 39.5 °C. Blood pressure, respiration and heart rate were within normal limits and physical examination was negative for an infectious focus. Laboratory parameters revealed an ANC of < 100/μL and CRP of 21.1 mg/dl (normal: < 0.5 mg/dl). Blood cultures were obtained through the indwelling triple-lumen Broviac catheter, and the patient was started on empiric antibacterial therapy with piperacillin plus tazobactam (100 mg/kg TID) and gentamicin (4 mg/kg QD) per institutional standard operating procedure (Fig. 1). The next day, *Gram-*negative coccoid bacteria were found in the blood cultures after overnight incubation at 37 °C. Grey-white colored colonies grew aerobically on blood agar. These were identified by matrix assisted laser desorption ionization-time of flight mass spectrometry (MALDI-TOF MS) as *Moraxella nonliquefaciens*. Given the lack of specific breakpoints for this microorganism, the agar diffusion test was interpreted according to the clinical breakpoints of the European Committee on Antimicrobial Susceptibility Testing for *M. catarrhalis* [14], confirming susceptibility to piperacillin/tazobactam. On day + 7, while still febrile, the patient developed low blood pressure (minimum mean arterial pressure: 51 mmHg), an increased heart rate (maximum: 160 beats/minute) and decreasing oxygen saturation (minimum: 89% at room air). Physical examination revealed an ill-appearing child with prolonged capillary refill but no apparent infectious focus. Appropriate supportive measures were initiated and the indwelling Broviac central venous catheter was surgically removed for source control, resulting in prompt circulatory stabilization.

During the following days the patient had recurrent fever of up to 39.0 °C and intermittent decreases in oxygen saturation during night time under continuous oxygen substitution. Daily follow-up blood cultures remained negative. A computed tomography of the chest revealed no abnormality, and clinical symptoms were attributed to grade 3 mucositis. Due to persistent fever with the possibility of a second invasive infection in the state of profound neutropenia, antibacterial therapy was escalated per institutional standard to meropenem (20 mg/kg TID) plus teicoplanin (10 mg/kg QD; day 1: 10 mg/kg BID), and fluconazole (12 mg/kg QD) was started to prevent mucosal and invasive candidiasis.

Following neutrophil recovery with formal neutrophil engraftment on day + 14, the patient’s general condition markedly improved and definite defervescence was noted on day + 17. Antibacterial therapy with meropenem plus teicoplanin was continued for a total of 11 days of appropriate therapy, and the patient was discharged after resolution of mucositis on day + 22 post stem cell infusion. Five months later the patient showed no signs of infection relapse.

## Discussion and conclusions

The family *Moraxellaceae* is a member of the order Pseudomonadales, consists of aerobic, Gram*-*negative, catalase positive, nonfermenting bacteria and is subdivided into three well-recognized genera, *Moraxella*, *Acinetobacter,* and *Psychrobacter* [15]. The genus *Moraxella* includes seven species, the most common of which is *Moraxella catarrhalis,* a frequent cause of otitis media in children and of infectious exacerbations of chronic obstructive pulmonary disease in adults [15, 16].

*M. nonliquefaciens* is a usually non-pathogenic microorganism that exists as part of the upper respiratory tract flora [1]. It has been reported as an occasional cause of localized invasive infections, including meningitis [2], endophthalmitis [3, 4], endocarditis [5–7], pneumonia [8], and septic arthritis [17]. Immediately life-threating conditions in the form of bloodstream infections seem to be even more rare: Including the case presented here, only five cases of *M. nonliquefaciens* bacteremia have been reported in the literature to date [9–12] (Table 1). In all cases, underlying diseases may have acted as predisposing factors: three patients presented with hemato-oncological disorders and treatment-induced neutropenia, and the remaining two patients had significant comorbidities. This suggests that *M. nonliquefaciens* has a relevant pathogenicity potential in immunocrompromised and critically ill patients, while it is unlikely to cause disease in healthy hosts. Bloodstream-associated and catheter-associated infections with other *Moraxella* species have been reported in cancer patients in association with chemotherapy-related mucositis [18], which constitutes a plausible portal of entry also in our patient.

The ability to produce biofilms observed in *M. nonliquefaciens* and other *Moraxella* species [15] and the interaction with other microorganisms within this milieu can contribute to bacterial persistence and resistance against antibiotic treatment [19]. In spite of the lack of specific susceptibility breakpoints for *M. nonliquefaciens*, an in vitro resistance pattern frequent in the genus *Moraxella* was observed, with resistance against penicillin, amoxicillin, ampicillin und piperacillin and susceptibility to piperacillin plus tazobactam that suggest the production of β-lactamases [20]. Although not possible to prove, and based on the evidence regarding the pathogenicity of *M. nonliquefaciens* in immunocompromised patients, the clinical deterioration of our patient at day two of appropriate antibacterial therapy with prompt recovery after removal of the indwelling central venous catheter suggests a role of biofilm in the pathogenesis of the evolving sepsis. This supports the importance of immediate source control for successful management of bloodstream infections by *M. nonliquefaciens.*

Although very infrequently, *Moraxella nonliquefaciens* may cause bloodstream infections, especially in immunocompromised patients. A successful therapeutic approach should include prompt source control and administration of adequate antibiotic therapy.

## Data Availability

Not applicable.

## Abbreviations

ANC: absolute neutrophil count
BID: twice a day
CRP: C-reactive protein
QD: once daily
TID: three times a day
